# Angiotensin Converting Enzyme Gene Insertion/Deletion Polymorphism and Vesicoureteral Reflux in Children: A Meta-Analysis of 14 Case–Control Studies

**DOI:** 10.1097/MD.0000000000002421

**Published:** 2015-12-31

**Authors:** Jin-Wei Ai, Yu Liu, Xian-Tao Zeng, Qing Lei, Li Zou, Bin Pei

**Affiliations:** From the Evidence-Based Medicine Center, Xiangyang Hospital, Hubei University of Medicine, Xiangyang, China (J-WA, YL, QL, LZ, BP); Department of Urology, Center for Evidence-Based Medicine and Translational Medicine, Zhongnan Hospital of Wuhan University, Wuhan, China (X-TZ).

## Abstract

Vesicoureteral reflux (VUR) is a common and serious urinary disease in children. It usually causes renal scar, urinary tract infection, and chronic renal failure. Previous studies showed the angiotensin converting enzyme (ACE) gene insertion/deletion (I/D) polymorphism might be associated with VUR; however, the conclusions were inconsistent. Therefore we used the meta-analytic approach to clarify the effect of ACE I/D polymorphism on VUR risk.

We systematically searched the PubMed, CNKI, and EMBASE databases to identify all the potentially related studies published up to February 4, 2015. Two reviewers independently selected studies and extracted data. The strength of the association was assessed using odd ratio (OR) with its 95% confidence interval (CI) based on fixed or random effects model. The STATA 12.0 software was used for data analysis.

A total of 14 case–control studies involving 1197 VUR patients and 1320 healthy controls met the eligibility criteria. Results of meta-analysis showed significant association between ACE I/D polymorphism and VUR risk (D vs. I: OR = 1.28, 95% CI = 1.06–1.54, *P* = 0.01; DD vs. II: OR = 1.44, 95% CI = 1.12–1.85, *P* = 0.01; DD vs. DI + II: OR = 1.49, 95% CI = 1.23–1.79, *P* < 0.01; DD + DI vs. II: OR = 1.20, 95% CI = 0.84–1.72, *P* = 0.31). Subgroup analyses revealed varied results. In Turkish people, results of all the genetic models other than DI vs. II showed statistical significance; in Caucasians, DD vs. DI + II showed statistical significance; and in Asians, DI versus II showed statistical significance.

Our meta-analysis indicated that the ACE I/D polymorphism might be associated with increased risk of VUR in children. However, due to the limitations, we suggest conducting additional studies with larger sample size and adjustment for various risk factors, in the future for further clarification.

## Abstract 2

Supplemental Digital Content is available in the text

## INTRODUCTION

Vesicoureteral reflux (VUR) is a common and serious urinary disease in children.^1^ Epidemiological studies indicated that the morbidity of VUR in children is 1% to 2%,^2^ which results in urinary tract infection (UTI) in 30% to 40% of the affected patients.^3^ Complicating hypertension, renal scar, reflux nephropathy (RN), end-stage renal disease (ESRD), and chronic renal failure (CRF) may develop during its progression.^4–6^ The VUR is a serious threat to adolescents’ health. Over the past 3 decades, it has been considered that genetic predisposition may play an important role in the development of VUR, and the angiotensin converting enzyme (ACE) gene insertion/deletion (I/D) polymorphism was one of the most frequently investigated.^7^

Human ACE gene is located on the chromosome 17q23. It spans 21 kb and is composed of 26 exons and 25 introns.^8^ The most common genetic variation is the I/D of a 287 bp Alu repetitive sequence in intron 16.^9^ There are 2 alleles (I and D) and 3 genotypes (II, DI, and DD).^9^ Previous studies indicated that the ACE DD genotype and/or D allele increased the risk of various renal diseases.^10–12^ However, there has always been a controversy pertaining to the association between ACE I/D polymorphism and the VUR susceptibility. Some studies suggested that ACE DD genotype increased the VUR risk, but others showed that there was no significant association.

One standard meta-analysis, which included 10 articles with 757 cases and 1066 controls, concluded that the ACE I/D polymorphism was not related to the risk of VUR in Caucasians and Asians, but DD genotype and D allele increased VUR risk in Turks.^13^ However, there was only one Turkish study in this meta-analysis.^13^ In addition, the study sample size was relatively small and obvious publication bias was detected, which indicated low reliability of its results. Therefore, we performed an updated meta-analysis including all eligible studies to provide a more robust verdict on the association between the ACE I/D polymorphism and VUR risk.

## METHODS

This meta-analysis was reported according to the PRISMA guidelines.^14^ The ethnic review was approved by the Xiangyang Hospital, Hubei University of Medicine.

### Eligibility Criteria

Studies met the following inclusion criteria were included: case–control or cohort studies; investigating the association between ACE I/D polymorphism and VUR risk in children; diagnostic imaging techniques such as renal ultrasonography, voiding cystourethrography, or nuclear scan with technetium-99m-dimercaptosuccinic acid were used for the diagnosis of VUR; healthy children as the control group; and with sufficient data for calculating the odds ratio (OR) and it 95% confidence interval (CI). Besides, editorials, duplicated reports and animal or cell line studies were excluded.

### Literature Search

We systematically searched the PubMed, CNKI (China National Knowledge Infrastructure), and Embase databases up to February 4, 2015 to identify all related studies. The medical subject headings (MeSH) and free text words were used. We combined search terms for VUR, ACE, and Genetic polymorphism. Search terms mainly included (vesico-ureteral reflux OR vesco-uretric reflux OR VUR) AND (peptidyl-dipeptidase A OR angiotensin converting enzyme OR ACE) AND (genetic polymorphism OR genetic variation). The detailed search strategy was shown in File S1. No language or other restrictions were imposed. Furthermore, we also hand-searched the reference list of all the retrieved studies and searched Google scholar to identify additional records, which were not included in those databases.

### Data Extraction

Two investigators independently selected the studies from which data of the following items were extracted: surname of first author, year of publication, study design, source of cases and controls, number of cases and controls, average age of cases and controls, ethnicity, genotyping method, VUR diagnostic method, genotype distribution of cases and controls, and Hardy–Weinberg equilibrium (HWE) in controls. Discussions aimed to resolve discrepancies by reaching consensus were held.

### Quality Assessment

Two investigators independently evaluated the quality of eligible studies using the Newcastle-Ottawa Scale (NOS), which was one of the most commonly used tools for assessing observational quality in a meta-analysis. The NOS included 3 parts, case and control selection, comparability, and exposure. Each of them respectively comprised 4, 2, and 3 items. What is more, we added an item “conform to HWE” to “case and control selection.” So, each item is given 1 point, 10 points in total. If less than 8 scores the study got, it would be regarded as “low quality”; otherwise, the study would be regarded as “high quality." In the case of any conflict, a discussion was initiated in order to arrive at a consensus.

### Data Analysis

Extracted data were loaded into STATA12.0 (Stata Corporation, College Station, TX) and analyzed. The OR and corresponding 95% CI were used to measure the strength of the association, and 5 common genetic models were used: D versus I, DD versus II, DD versus DI, DD versus DI + II, and DD + DI versus II. The heterogeneity was measured using the I^2^ statistic and Cochran Q test before performing pooled analysis. When I^2^ < 50% and *P* > 0.1, we chose the fixed-effects model, otherwise the random-effects model was chosen. The statistical significance of the pooled ORs was judged using a 2-tailed *P*-values (*P* < 0.05 was deemed statistically significant). Subgroup analyses stratified by ethnicity and HWE status were performed. Sensitivity analyses were performed by sequentially excluding each single study. Funnel plots and Egger test were used to evaluate the publication bias.

## RESULTS

### Study Selection

**FIGURE 1:**
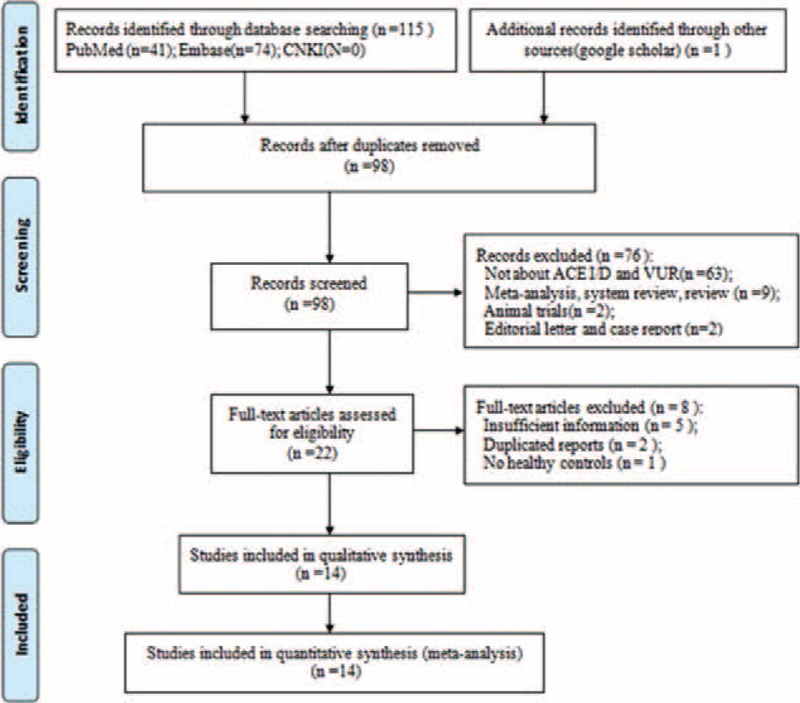
Flow diagram of the selection process for eligible studies.

Figure 1 summarizes the detailed process of study selection. A total of 116 articles were identified by the literature search. After the titles and abstracts were reviewed, 22 studies were processed to further full-text selection, through which 8 papers were excluded. Of these papers, five^15–19^ were ruled out due to insufficient information about ACE I/D genotypes, one^20^ due to lack of healthy controls, and the other two^21,22^ were duplicated reports. Finally, a total of 14 case–control studies,^23–36^ with 1197 VUR patients and 1320 healthy controls, were included in our meta-analysis.

### Characteristics of Included Studies

Table 1 shows the essential characteristics of the included studies, as well as genotype distributions, HWE status, and quality assessment. All these studies were published in English. Four^33–36^ of these studies were performed in Turks, seven^23–29^ in Caucasians, and three^30–32^ in Asians. Only healthy individuals were recruited as the control group for each study. Moreover, the Polymerase Chain Reaction (PCR) technique was used for genotyping, and the diagnosis of VUR was based on diagnostic imaging techniques including renal ultrasonography, voiding cystourethrography, and/or nuclear scan with technetium-99m-dimercaptosuccinic acid. The ACE D allele's average frequency in the cases and controls was respectively 56.0% and 51.0%, and was 60.2%, 57.1%, and 37.5% respectively in Turks, Caucasians, and Asians. The ratio between cases and controls for the mean frequency of D allele in Turks, Caucasians, and Asians was 1.20, 1.10, and 1.15, respectively. The controls’ genotype distribution conformed to HWE in all but 1 study.^33^ The qualities of primary studies assessed by NOS. Only 4 studies got 7 score, others more than 7. In other words, 4 studies were regarded as “low quality,” and 10 as “high quality.” The average score was 8.07, which indicated that overall quality of the studies was high. The detailed quality assessment was shown in File S2.

**TABLE 1:**
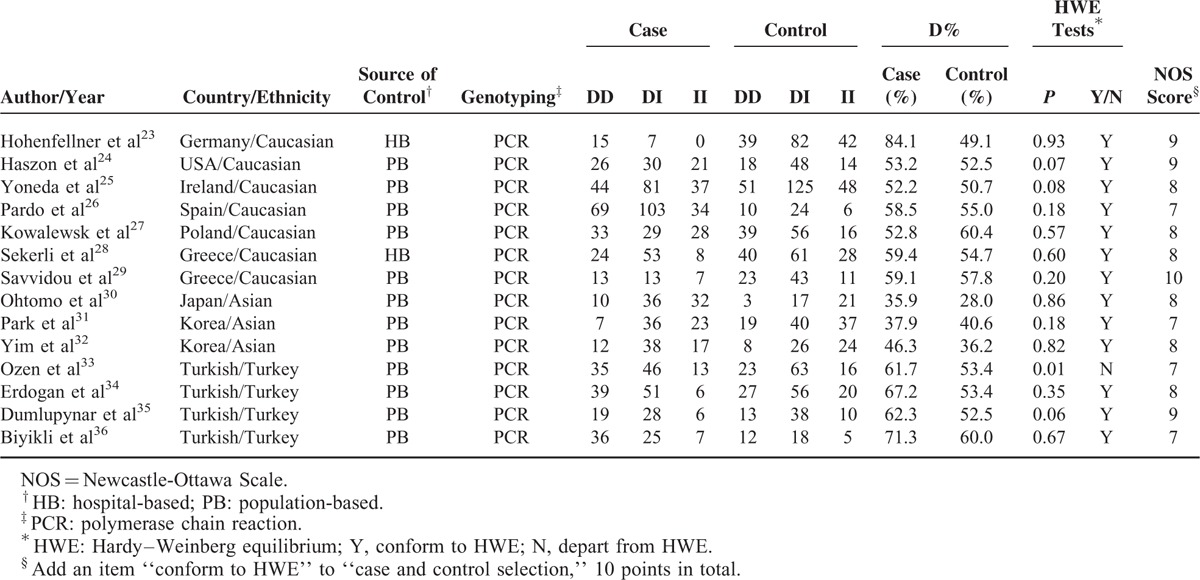
Characteristics of the Studies Included in This Meta-Analysis

### Meta-Analysis

Tables 2 and 3 present a summary of results of meta-analysis and subgroup analysis concerning the association between ACE I/D polymorphism and VUR risk.

**TABLE 2:**
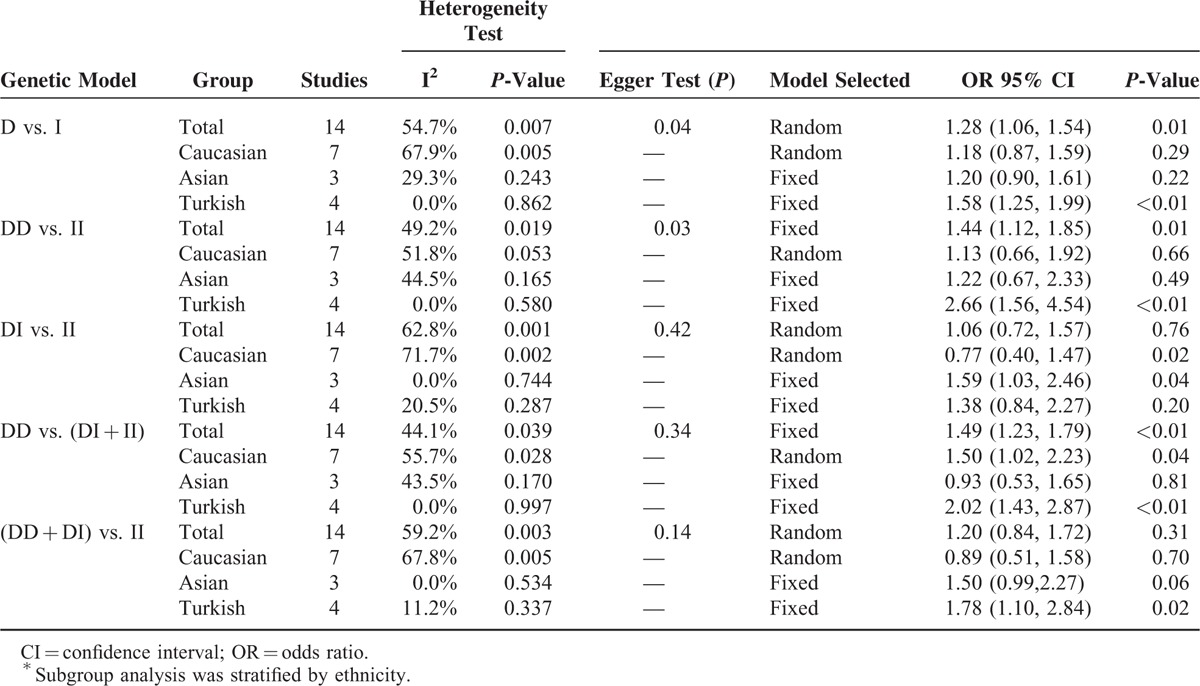
A Summary of the Meta-Analysis and Subgroup Analysis^∗^

**TABLE 3:**
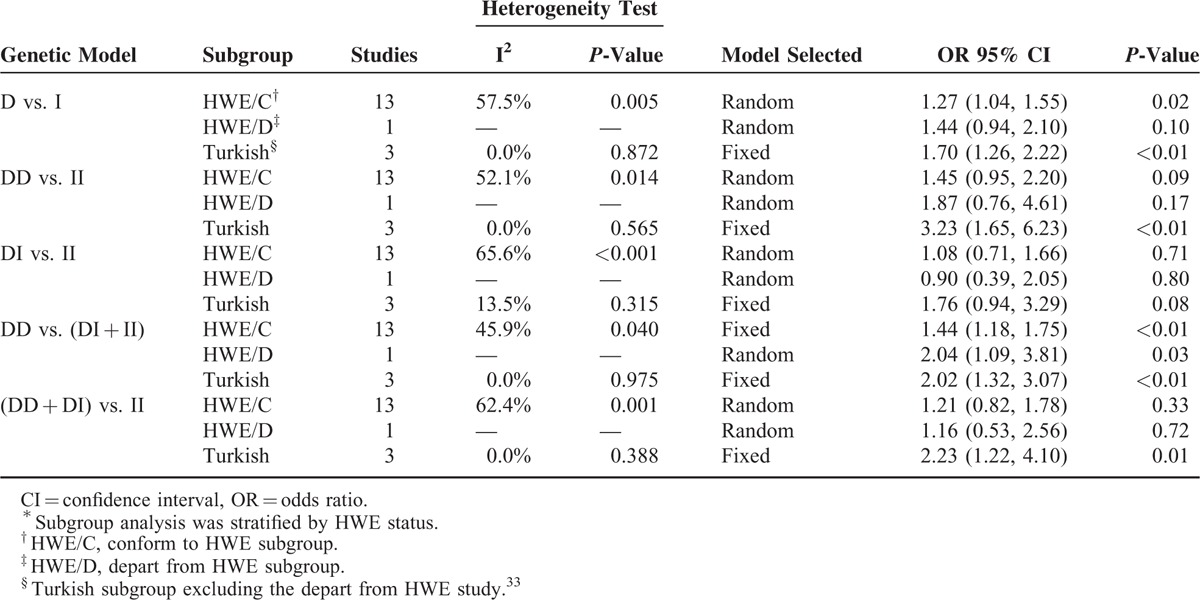
Subgroup Analysis^∗^

**FIGURE 2:**
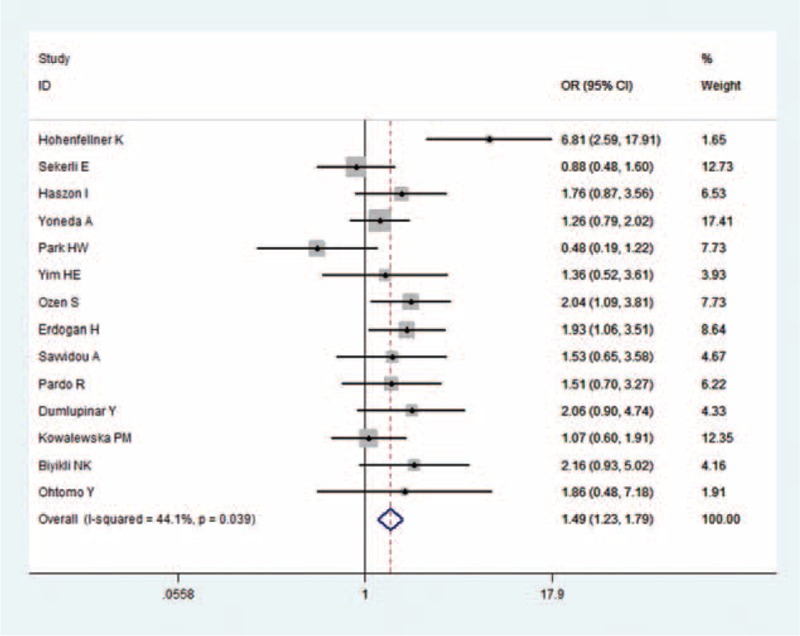
Meta-analysis for ACE I/D polymorphism and VUR in children under the DD vs. DI + II genetic model.

The pooled ORs of all 14 case–control studies revealed that the ACE I/D polymorphism was significantly associated with increased risk of VUR: D versus I: OR = 1.28, 95% CI = 1.06–1.54, *P* = 0.01; DD versus II: OR = 1.44, 95% CI = 1.12–1.85, *P* = 0.01; DD versus DI + II: OR = 1.49, 95% CI = 1.23–1.79, *P* < 0.01, Figure 2; and DD + DI versus II: OR = 1.20, 95% CI = 0.84–1.72, *P* = 0.31.

Subgroup analyses stratified by ethnicity suggested the association between ACE I/D polymorphism and VUR risk were different among different races and genetic models. In Turks, the results showed that the ACE DD genotype and D allele increased the risk of VUR (Table 2). In Caucasians, DD versus DI + II: OR = 1.50, 95% CI = 1.02–2.23, *P* = 0.04. In Asians, DI versus II: OR = 1.59, 95% CI = 1.03–2.46, *P* = 0.04.

As to stratified analysis by HWE status, the results also indicated that ACE DD genotype and/or D allele increased the risk of VUR in children. In the subgroup conforming to HWE, D versus I: OR = 1.27, 95% CI = 1.04–1.55, *P* = 0.02; and DD versus DI + II: OR = 1.44, 95% CI = 1.18–1.75, *P* < 0.01. In the subgroup inconsistent with HWE, DD versus DI + II: OR = 2.04, 95% CI = 1.09–3.81, *P* = 0.03. Because the non-HWE study came from the Turkish group, we recalculated the pooled effects of Turkish group without considering the very study, which did not result in significant change (Table 3).

**FIGURE 3:**
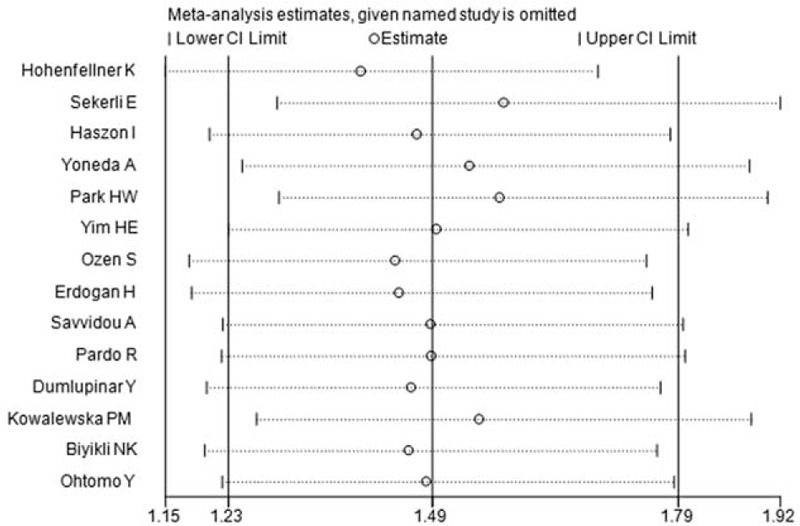
Sensitivity analysis of included studies, (DD vs. DI+II genetic model).

We performed sensitivity analysis by excluding each single study sequentially. The results were not significantly altered except for omitting the study of Ozen et al^33^ under DD versus II genetic model, as previously mentioned (Figure 3).

### Publication Bias

**FIGURE 4:**
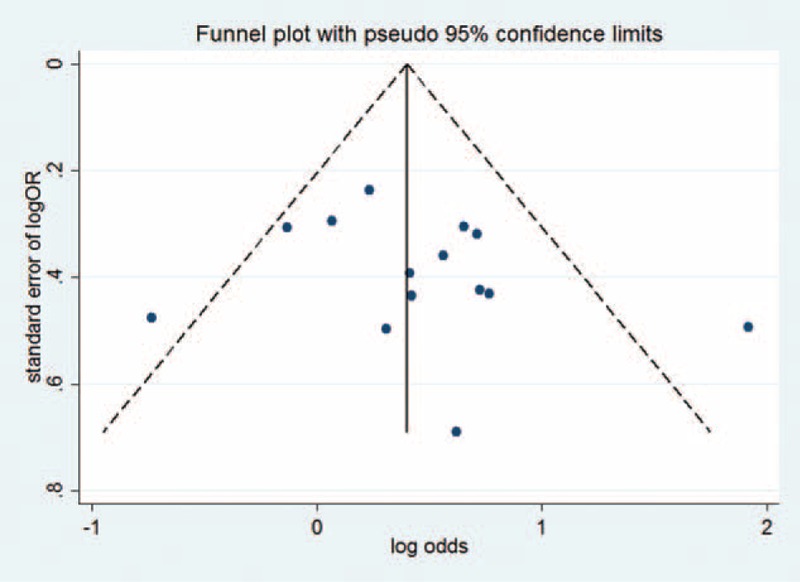
Funnel plot to detect publication bias (DD vs. DI + II genetic model).

The shape of the funnel plot did not reveal any evidence of funnel plot asymmetry (Figure 4 displayed a funnel plot for DD vs. DI + II genetic model). But the statistical results shown there were publication bias in D versus I and DD versus II genetic models (Table 2).

## DISCUSSION

This meta-analysis based on 14 case–control studies involving 1197 VUR cases and 1320 healthy controls indicated that ACE DD genotype and D allele were associated with increased risk of VUR in the overall population. There were mild to moderate heterogeneity across included studies, and the heterogeneity may be attributable to ethnic variation, because the average frequency of D allele was notably different in different races. The average frequency of D allele in Turks was 60.2%, in Caucasians was 57.1%, and in Asians was 37.5%. So, we performed a subgroup analysis by ethnicity, and the results suggested the ACE I/D polymorphism was significantly associated with VUR risk in Turks and Caucasians, but not in Asians.

In Turkish subgroup, the DD genotype and D allele increased the risk of VUR. The heterogeneity for this subgroup analysis were tiny, with I^2^ = 11.2% in DD + DI versus II, I^2^ = 20.5% in DI versus II, and I^2^ = 0% in all the other genetic models. No significant change was found in sensitivity analysis. Therefore, the conclusions of the Turkish subgroup were very reliable. In Caucasians, we found the DD genotype increased the VUR risk. There was moderate heterogeneity across Caucasian studies. Although we failed to explore the source of heterogeneity due to the insufficient data acquired from the original researches, the large study sample size along with the result stability revealed by sensitivity analysis indicated that the results of Caucasians were relatively dependable. In Asians, DI versus II genetic models showed statistical significance, indicating the DI genotype increased the risk of VUR in Asian children as compared to II. The heterogeneity in this genetic model was tiny (I^2^ = 0%), and the results were not significantly changed in sensitivity analysis. So, the conclusion in Asians was also credible.

In HWE-consistent subgroup, the pooled results also suggested that ACE DD genotype and D allele were risk factors for VUR in children. In sensitivity analysis, when we excluded the study of Ozen et al,^33^ in which the controls’ genotype distribution departed from HWE, the pooled result was significantly changed in DD versus II genetic model. It only suggested that the DD carriers, compared with II, increased the VUR risk, and further investigation is needed. This change did not affect our conclusions that DD genotype, compared with DI or DI + II, increased the VUR susceptibility. In particular, the pooled results were not changed in the Turkish subgroup, where the study came from. So, the departure from HWE's study did not affect the results of our meta-analysis.

The Egger test indicated that our included studies had publication bias in D versus I and DD versus II genetic models. But the shape of the funnel plots was not obviously asymmetry (Figure S1 and S2). We also estimated the publication bias by Begg test (File S3). We found *P* > 0.05 in all genetic models, and this suggested our included studies might not have publication bias. By further analysis of the reasons for the contradictions of 2 tests, one study^23^ showed more obviously effect. When we excluded the study,^23^ the *P* values changed to >0.05 in Egger test (*P* = 0.28 and 0.15, respectively), without significant change of the pooled results, which was consistent with sensitivity analysis. So, the publication bias in 2 genetic models did not affect the reliability of our study results.

VUR is a complex urinary system disease with a wide range of risk factors.^7^ ACE I/D polymorphism as a genetic factor has been comprehensively investigated. But the exact nosogenesis underlying the relationship between the polymorphism and VUR was not completely understood. Previous studies demonstrated that ACE DD genotype enhanced the ACE expression.^37,38^ ACE is a key enzyme in the renin-angiotensin system (RAS). Subjects with the DD genotype have the highest tissue and plasma ACE level.^39^ ACE takes part in blood pressure, cardiovascular function, and electrolyte homeostasis regulation by facilitating the conversion of Angiotensin I (Ang I) into Angiotensin II (Ang II).^40^ Elevated Ang II are effective in the progression of renal disease, not just through hemodynamic effects but also through growth-related and prosclerotic effects.^41^ Angiotensin II binds to its receptors, that is, AT1 (Angiotensin II type 1 receptor) and AT2 (Angiotensin II type 2 receptor), and, through the activation of different intracellular signaling pathways, mediates the production of various profibrotic and proinflammatory factors, such as transforming growth factors, cytokines, chemokines, and adhesion molecules. The intrarenal concentration of Ang II in the ACE DD genotype is 1000 times higher than that of plasma.^28^ It increases the intraglomerular pressure, induces transforming growth factor to exert a prosclerotic activity leading to interstitial proliferation, and prevent the degradation of the glomerular interstitium, further aggravating glomerular sclerosis.^15^ Thus, the genetic polymorphism of the ACE I/D may be associated with the occurrence and progression of VUR. However, more experimental or clinical studies should be performed to explain the precise pathophysiologic mechanisms of the ACE DD genotype and D allele increasing the VUR risk.

In 2012, Zhou et al^13^ also performed a meta-analysis to explore the association between ACE I/D polymorphism and UVR risk. The most important advantage of our meta-analysis was that our results were not the same with this previously meta-analysis. The previous meta-analysis^13^ based on 10 articles with 757 cases and 1066 controls was included in this study, which concluded that the ACE I/D polymorphism was not related to the risk of VUR in the overall population, Caucasians and Asians, but DD genotype and D allele increased VUR risk in Turks. The study sample size was smaller than our meta-analysis. Although a significant association was revealed for the Turkish population, it should be noted that only one Turkish study was included in this meta-analysis.^13^ Moreover, this study^13^ had obvious publication bias. Therefore, we performed the updated meta-analysis with more eligible studies, and drew a more stable conclusion. As mentioned above, our results were distinctly different from Zhou et al,^13^ and we did many new discoveries. What is more, the publication bias in 2 genetic models did not affect the reliability of our study results. Thus, our results were more reliable with enlarged sample sizes.

Our study has 3 limitations. First, we were unable to carry out adjusted analysis for confounders such as gender and environment due to lack of relevant original data. As we know, different gender may have different genotype distribution, different environment may also appear different VUR incidence; however, we failed to perform further investigations for the gene–gene, gene–gender, and gene–environment interactions effect. Second, also due to original data limited, we were unable to explore the association between ACE I/D polymorphism and VUR reflux grades. Although many studies reported ACE DD genotype correlated to high grade of reflux, it was also controversial. We suggest further studies should report the VUR reflux grades and explore whether ACE I/D polymorphism is associated with VUR reflux grades. Finally, the number of included studies and involved sample size remain not large enough. Although we preformed subgroup analyses by ethnicity and HWE status, the heterogeneity could not be completely resolved.

In summary, the present meta-analysis demonstrated that the ACE DD genotype and D allele might be associated with increased risk of VUR in children. However, due to the limitation of the present studies, more well-designed large-scale investigations are warranted to further confirm our findings.

## Supplementary Material

Supplemental Digital Content
