# Supplementary material for: Angiotensin Converting Enzyme Gene Insertion/Deletion Polymorphism and Vesicoureteral Reflux in Children: A Meta-Analysis of 14 Case–Control Studies
Source: Medicine (Baltimore). 2015 Dec 31;94(52):e2421. doi: 10.1097/MD.0000000000002421 (PMC5291643; doi:10.1097/MD.0000000000002421)
Supplement: Supplemental Digital Content [file medi-94-e2421-s001.doc]

File S1. The detailed search strategy in PubMed

#1 Search "Vesico-Ureteral Reflux"[Mesh]

#2 Search (((((("vesico-ureteral reflux") OR "vesico ureteral reflux") OR "vesco-uretric reflux") OR "vesco uretric reflux") OR "reflux nephropathy") OR "vesicoureteral reflux") OR VUR

#3 #1 OR #2

#4 Search "Peptidyl-Dipeptidase A"[Mesh]

#5 Search (((Angiotensin) OR Angiotensins) OR "angiotensin converting enzyme") OR ACE

#6 #4 OR #5

#7 Search "Polymorphism, Genetic"[Mesh]

#8 Search (((((("Genetic Polymorphism") OR "Genetic Polymorphisms") OR "Genetic Variation") OR polymorphism) OR polymorphisms) OR gene) OR genome

#9 #7 OR #8

#10 #3 AND #6 AND #9

The complete search strategy: (((("Vesico-Ureteral Reflux"[Mesh]) OR ((((((("vesico-ureteral reflux") OR "vesico ureteral reflux") OR "vesco-uretric reflux") OR "vesco uretric reflux") OR "reflux nephropathy") OR "vesicoureteral reflux") OR VUR))) AND (("Peptidyl-Dipeptidase A"[Mesh]) OR ((((Angiotensin) OR Angiotensins) OR "angiotensin converting enzyme") OR ACE))) AND (("Polymorphism, Genetic"[Mesh]) OR ((((((("Genetic Polymorphism") OR "Genetic Polymorphisms") OR "Genetic Variation") OR polymorphism) OR polymorphisms) OR gene) OR genome))

| File S2. The quality assessment of the included studies(Newcastle-Ottawa Scale, NOS)^†^ | | | | | | | | | | | | | |
| --- | --- | --- | --- | --- | --- | --- | --- | --- | --- | --- | --- | --- | --- |
| Study ID | Case and control selection | | | | |  | Comparability | |  | Exposure | | | Total  Score |
|  | Case definition | Case representativeness | Controls selection | Conform to HWE | Controls definition |  | Control for race | Control for others^Δ^ |  | Accuracy | Uniformity | Non-Response |  |
| Hohenfellner 1999 | 1 | 1 | 1 | 1 | 1 |  | 1 | 0 |  | 1 | 1 | 1 | 9 |
| Haszon 2002 | 1 | 1 | 1 | 1 | 1 |  | 1 | 0 |  | 1 | 1 | 1 | 9 |
| Yoneda 2002 | 1 | 1 | 0 | 1 | 1 |  | 1 | 0 |  | 1 | 1 | 1 | 8 |
| Pardo 2003 | 1 | 1 | 0 | 1 | 0 |  | 1 | 0 |  | 1 | 1 | 1 | 7 |
| Kowalewska 2004 | 1 | 1 | 1 | 1 | 0 |  | 1 | 0 |  | 1 | 1 | 1 | 8 |
| Sekerli 2009 | 1 | 1 | 0 | 1 | 1 |  | 1 | 0 |  | 1 | 1 | 1 | 8 |
| Savvidou 2010 | 1 | 1 | 1 | 1 | 1 |  | 1 | 1 |  | 1 | 1 | 1 | 10 |
| Ohtomo 2001 | 1 | 1 | 1 | 1 | 0 |  | 1 | 0 |  | 1 | 1 | 1 | 8 |
| Park 2000 | 1 | 1 | 0 | 1 | 0 |  | 1 | 0 |  | 1 | 1 | 1 | 7 |
| Yim 2004 | 1 | 1 | 0 | 1 | 1 |  | 1 | 0 |  | 1 | 1 | 1 | 8 |
| Ozen 1999 | 1 | 1 | 0 | 0 | 1 |  | 1 | 0 |  | 1 | 1 | 1 | 7 |
| Erdogan 2004 | 1 | 1 | 0 | 1 | 1 |  | 1 | 0 |  | 1 | 1 | 1 | 8 |
| Dumlupinar 2010 | 1 | 1 | 1 | 1 | 1 |  | 1 | 0 |  | 1 | 1 | 1 | 9 |
| Biyikli 2007 | 1 | 1 | 0 | 1 | 0 |  | 1 | 0 |  | 1 | 1 | 1 | 7 |
| ^†^, add an item “conform to HWE” to “case and control selection”, 10 points in total; ^Δ^, Control for other additional factors(e.g., age and gender, etc.) | | | | | | | | | | | | | |
